# d-xylose accelerated death of pentose metabolizing *Saccharomyces cerevisiae*

**DOI:** 10.1186/s13068-023-02320-4

**Published:** 2023-04-17

**Authors:** Jeroen G. Nijland, Xiaohuan Zhang, Arnold J. M. Driessen

**Affiliations:** Molecular Microbiology, Groningen Biomolecular Sciences and Biotechnology, Nijenborgh 7, 9747AG Groningen, The Netherlands

**Keywords:** d-xylose consumption, ATP, Xks1 expression, Bioethanol, Yeast

## Abstract

Rapid and effective consumption of d-xylose by *Saccharomyces cerevisiae* is essential for cost-efficient cellulosic bioethanol production. Hence, heterologous d-xylose metabolic pathways have been introduced into *S. cerevisiae*. An effective solution is based on a xylose isomerase in combination with the overexpression of the xylulose kinase (Xks1) and all genes of the non-oxidative branch of the pentose phosphate pathway. Although this strain is capable of consuming d-xylose, growth inhibition occurs at higher d-xylose concentrations, even abolishing growth completely at 8% d-xylose. The decreased growth rates are accompanied by significantly decreased ATP levels. A key ATP-utilizing step in d-xylose metabolism is the phosphorylation of d-xylulose by Xks1. Replacement of the constitutive promoter of *XKS1* by the galactose tunable promoter Pgal10 allowed the controlled expression of this gene over a broad range. By decreasing the expression levels of *XKS1*, growth at high d-xylose concentrations could be restored concomitantly with increased ATP levels and high rates of xylose metabolism. These data show that in fermentations with high d-xylose concentrations, too high levels of Xks1 cause a major drain on the cellular ATP levels thereby reducing the growth rate, ultimately causing substrate accelerated death. Hence, expression levels of *XKS1* in *S. cerevisiae* needs to be tailored for the specific growth conditions and robust d-xylose metabolism.

**Supplementary Information:**

The online version contains supplementary material available at 10.1186/s13068-023-02320-4.

## Introduction

In a world where energy from fossil fuels is less desired, the production of liquid fuels from renewable feedstocks has been stimulated and researched intensively. Bioethanol, currently mainly used as an additive to fuel, is produced from agricultural feedstocks like sugar cane and corn which are readily fermentable. This so-called first generation biofuel process is unfavorable because the production of the required feedstock’s competes, using large amounts of arable land, with the food supply [1]. The second generation biofuel process uses a more sustainable source of feedstock since the required lignocellulosic biomass is obtained from agricultural waste material [2]. However, a major drawback of lignocellulosic feedstocks is the inability of *Saccharomyces cerevisiae*, the most commonly used yeast in the bioethanol industry, to ferment pentose sugars, such as d-xylose. Lignocellulosic feedstock’s contains, next to hexose sugars, a substantial fraction of d-xylose (up to ~ 30%, [3]) that is released upon conversion of lignocellulose [4]. To convert d-xylose into bioethanol two different pathways have been integrated and optimized in *S. cerevisiae*: (1) the XR-XDH pathway, a two-step redox pathway in which d-xylose is reduced to xylitol by xylose reductase (XR) and subsequently the xylitol is oxidized by xylitol dehydrogenase (XDH) to form d-xylulose [5–8] and (2) the XI pathway, in which d-xylose is directly converted into d-xylulose using either a bacterial or fungal xylose isomerase [9–13]. In the current study the fungal xylose isomerase of *Piromyces *sp. E2 is used which is overexpressed using the Tpi1 promotor [14] and is present in nine genomic copies [15]. d-xylulose is subsequently phosphorylated by the xylulose kinase Xks1, which has been overexpressed in many engineered strains [12, 16, 17] and which is overexpressed using the Tef1 promotor in the currently used d-xylose-fermenting strain [15]. The resulting d-xylulose-5-phosphate enters the pentose phosphate pathway (PPP) and, via d-glyceraldehyde-3-phosphate and d-fructose-6-phosphate, d-xylose catabolism is connected to glycolysis. Various genetic modifications have improved d-xylose consumption e.g. via the deletion of *GRE3* [18–20] and the deletion of *PMR1* in IMX730 [21]. To further improve d-xylose consumption, numerous studies have overexpressed all involved genes in d-xylose metabolism (including the pentose phosphate pathway) at various levels, using (1) different promoters, and (2) increased genomic copy numbers. Xks1 which converts d-xylulose into d-xylulose-5-phosphate at the expense of one ATP, is one of the proteins that generally is overexpressed at high levels. Furthermore, rapid d-xylose consumption requires d-xylose transport into the cell which in *S. cerevisiae* is mediated via the large family of hexose transporters (HXT) of which eight are highly expressed depending on the carbon source (and concentration thereof) [22–24]. Although d-xylose enters cells via the HXT transporters, the affinity for d-glucose is in general 10–100 times higher as compared to d-xylose [25–27]. Remarkably, various studies have shown that the V_max_ for d-xylose of many HXT transporters is comparable to [28–30], or is even higher (e.g. Hxt1 [30] or Gal2 [31]) than that of d-glucose. Due to the HXT redundancy and the high transport rates in *S. cerevisiae*, we assume that in a strain containing all HXT transporters the d-xylose transporting capacity, in the absence of glucose [29, 31] and at higher d-xylose concentrations [32], is not a rate limiting step in d-xylose metabolism. The d-xylose metabolism rate could potentially also be determined by the xylose isomerase, however, the d-xylose-consuming strain used in the current study, and in most other studies, contains multiple copies of the xylose isomerase of *Piromyces *sp. E2 which is constitutively expressed via the strong Tpi1 promoter [15]. Furthermore, previous studies have suggested that the low metabolic activity of glycolysis [33] or more specifically, the lower part of glycolysis [34] are potential limiting factors in d-xylose metabolism.

Here we report that in a d-xylose metabolism engineered yeast strain, growth inhibition occurs at high d-xylose concentrations. By studying the dependence of high-performance d-xylose metabolism on the d-xylulose kinase Xks1, we show that d-xylose accelerated death can be prevented by a more balanced Xks1 gene expression.

## Materials and methods

### Yeast stains, media and culture conditions

The IMX730 xylose-fermenting *S. cerevisiae* strain (Additional file 1: Table S1), used in this study, was provided by Prof. Jack T Pronk, Department of Biotechnology, Delft University of Technology [15]. Aerobic shake flask and aerobic 96 wells micro-titer plates experiments were performed at 200 rpm in mineral medium (MM) supplemented with vitamin solution, urea (2.3 g/L), trace elements and d-xylose and/or d-glucose [35]. The composition of MM is as follows: K_2_SO_4_, 6.6 g/L; KH_2_PO_4_, 3 g/L; and MgSO_4_·7H_2_O, 0.5 g/L. The composition of trace elements is as follows: EDTA, 15 mg/L; ZnSO_4_·7H_2_O, 4.5 mg/L; CoCl_2_·6H_2_O, 0.3 mg/L; MnCl_2_·2H_2_O, 0.84 mg/L; CuSO_4_·5H_2_O, 0.3 mg/L; CaCl_2_·2H_2_O, 4.5 mg/L; FeSO_4_·7H_2_O, 3.0 mg/L; Na_2_MoO_4_·2H_2_O, 0.4 mg/L; H_3_BO_3_, 1.0 mg/L; and KI, 0.1 mg/L. The composition of vitamin solution is as follows: biotin (C_10_H_16_N_2_O_3_S), 0.05 mg/L; calcium pantothenate (C_18_H_32_CaN_2_O_10_), 1.0 mg/L; nicotinic acid (C_6_H_5_NO_2_), 1.0 mg/L; *myo*-inositol (C_6_H12O_6_), 25.0 mg/L; thiamine-HCl (C_12_H_18_C_l2_N_4_OS.xH_2_O), 1.0 mg/L; pyridoxol-HCl (C_8_H_12_ClNO_3_), 1.0 mg; and *para*-aminobenzoic acid (C_7_H_7_NO_2_), 0.2 mg/L. No silicone antifoam was used in any experiments and when applicable 20 mg/L uracil and 20 mg/L l-histidine was added. In all growth and ATP experiments a starting OD_600_ of 0.1 was used which was measured by optical density (OD) at 600 nm using an UV–visible spectrophotometer (Novaspec Plus, Amersham Biosciences).

### Strain construction

Strain IMX730 was used for further engineering to allow the controlled expression of the *XKS1* gene. To use histidine as autotrophic marker, the only available autotrophic marker, uracil, was used to delete the *Sp*his5 gene, using the Cas9 system. pMel10 [36], carrying the uracil autotrophic marker, was linearized by PCR using Phusion^®^ High-Fidelity PCR Master Mix in HF buffer (Thermo fisher scientific) and the *Sp*his5 specific target (Additional file 1: Table S2) was integrated using the Gibson Assembly^®^ Master Mix (New England Biolabs) which yielded Pmel10-His5 (Additional file 1: Table S1). After transformation [37] to the IMX730 strain using the pMel10-His5 plasmid and the his5 repair fragment (Additional file 1: Table S2), colonies, obtained on plates without uracil but with histidine, were selected using colony PCR with Phire® Green Hot Start II PCR Master Mix (Thermo fisher scientific) and the primers listed in Additional file 1: Table S2. This yielded IMX730△H (Additional file 1: Table S1) which was confirmed by the absence of growth in MM without histidine. In the same manner, using Cas9/pMel16, the promoter of the *XKS1* gene was replaced by the galactose inducible Gal10 promotor (451 bp) however the repair fragment, containing pGAL10, was amplified from genomic DNA from the original IMX730 strain. The targets and primers, to amplify the new fusion of the Gal10 promotor and the *XKS1* gene, are listed in Additional file 1: Table S2.

### ATP analysis

Intracellular ATP levels were analyzed using the BacTiter-Glo^™^ Microbial Cell Viability Assay (Promega) which allows for fast analysis without the isolation of intracellular metabolites. As described in the instruction from Promega, the BacTiter-Glo^™^ reagent lyses yeast cells to release the intracellular ATP. Subsequently, the luciferin in the BacTiter-Glo™ reagent reacts with ATP and O_2_ and is converted into oxyluciferin, which is detected by luminescence. All analyzed strains were grown for 16 h in MM containing 0.5% d-xylose (or otherwise as indicated), harvested by centrifugation at 2250 g, 25 ℃, and resuspended in MM at an OD_600_ of 0.2 without d-xylose. d-xylose was added at different concentrations and the ATP levels were measured in time by mixing 50 μL cell culture with 50 μL BacTiter-Glo^™^ Microbial Cell Viability Assay-Mix.

### RNA extraction, cDNA synthesis and RT-PCR

Total RNA was isolated from the engineered *S. cerevisiae* strains by a glass-bead disruption combined with a Trizol (Life Technologies) extraction procedure and cDNA was prepared as described previously [29]. The IMX730△H strain and IMX730-pGAL::XKS1 were inoculated, in duplicate, in MM containing 0.5% d-xylose and grown for 16 h. Subsequently, strains were diluted in the same medium to an OD_600_ of ~ 0.2 and grown for 3 h with the addition of 0.00312, 0.00625, 0.0125, 0.025, 0.05, 0.1, 0.2, 0.5 and 1.0% galactose before RNA was isolated. The expression of actin (*ACT1*) was used to normalize the various samples and the expression of the *GAL2* hexose transporter (as control for galactose inducibility) and *XKS1* was analyzed using the primers listed in Additional file 1: Table S3.

### Kinase activity analysis

Cells were grown, in MM complemented with 1% ethanol, for 16 h and subsequently diluted to an OD_600_ of 0.5 in MM with 0.5% ethanol and 0, 0.025, 1% galactose. After 3 h of induction 5 mL of the cell culture was centrifuged (3 min 2250*g*) and cells were resuspended in 500 μL MM and cell free extracted (CFE) was isolated by glass-bead disruption. Subsequently, the cell debris was centrifuged (2 min, 12,000*g*) and 2 μL of CFE was used in the Kinase Assay Kit (Sigma aldrich) with and without the addition of 6.66 mM (1 mg/mL) d-xylulose and 0.1 mM ATP. The CFE/D-xylulose/ATP/kinase assay mixture was incubated for 20 min and fluorescence was subsequently measured (at 590 nm) using the SynergyMx 96 wells plate reader (BioTek).

## Results

### Growth on various d-xylose concentrations

**Fig. 1:**
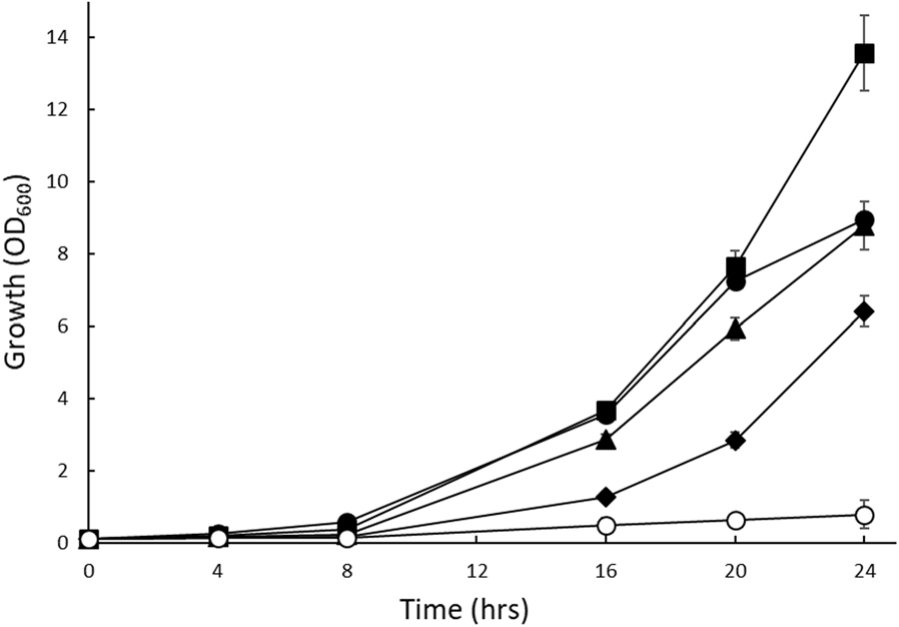
Aerobic growth of the IMX730-▲H strain in mineral medium containing 0.5% d-xylose (●), 1.0% d-xylose (■), 2.0% d-xylose (▲), 4.0% d-xylose (◆) and 8.0% d-xylose (○) complemented with l-histidine. Error bars were obtained from biological duplicates

Depending on the source, lignocellulosic biomass contains considerable amounts of d-xylose [3] which should be converted, at high rates and yields, into ethanol to establish an economically feasible industrial process. High concentrations of d-xylose should therefore be tolerated which was the starting point of an aerobic growth experiment with the IMX730△H strain in mineral medium containing 0.5 up to 8% d-xylose. This histidine dependent strain is derived from IMX730 which contains an engineered d-xylose metabolic pathway based on the fungal xylose isomerase of *Piromyces *sp. E2 and the overexpression of genes involved in the non-oxidative branch of the pentose phosphate pathway [15]. At d-xylose concentrations of 1% and below, the growth rates of IMX730△H were comparable but because of the lower d-xylose concentration, the total amount of biomass (OD_600_) decreased with the amount of d-xylose. Growth rates, however, deteriorated when the d-xylose concentration was increased to 2 and 4% while at 8% d-xylose hardly any growth could be observed after 24 h (Fig. 1).

The D-xylose consuming specialist strain IMX730△H is a quadruple hexo(gluco/galacto)kinase deletion strain (△Hxk1, △Hxk2, △Glk1 and △Gal1). To exclude the possibility that the absence these hexo(gluco/galacto)kinases influences the growth rates on high d-xylose concentrations, Hxk2 was expressed using plasmid pRS313-P7T7-Hxk2 [29]. Growth rates of IMX730-Hxk2 were similarly decreased at higher d-xylose concentration (Additional file 1: Figure S1A) as compared to the IMX730△H strain. In contrast, the growth rates of this strain on 8% d-glucose were not affected (Additional file 1: Figure S1B). Therefore, these data indicate that growth inhibition at high concentrations is specific for d-xylose and is not influenced by the presence of Hxk2.

### ATP levels in d-xylose metabolizing cells

**Fig. 2:**
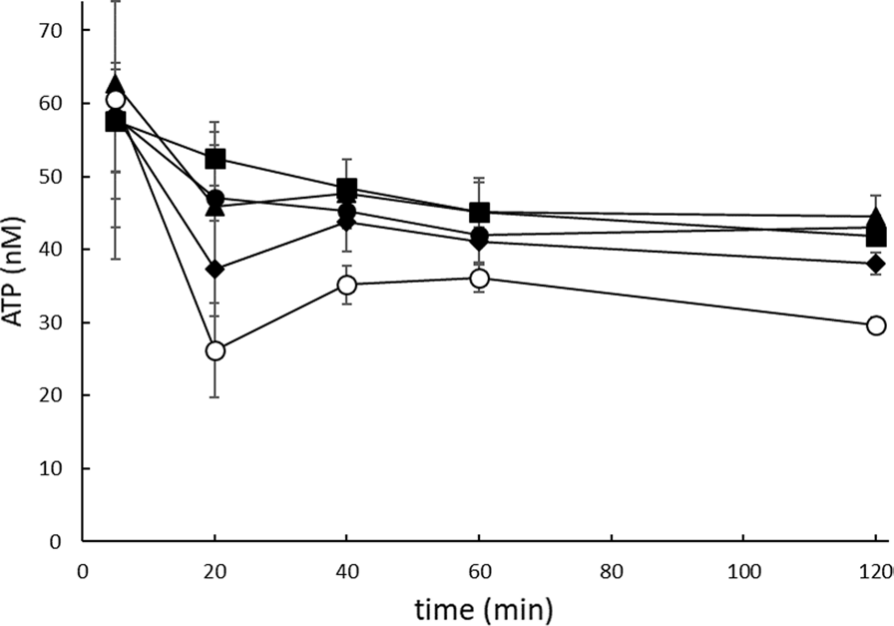
Intracellular ATP analysis after adding (T0) 0.5% d-xylose (●), 1.0% d-xylose (■), 2.0% d-xylose (▲), 4.0% d-xylose (◆) and 8.0% d-xylose (○) in the IMX730-△H strain. The IMX730-△H pre-culture was grown aerobically for 16 h in mineral medium supplemented with 0.5% d-xylose and l-histidine. Error bars were obtained from biological triplicates

d-glucose metabolism is, in contrast to d-xylose metabolism, regulated using the trehalose-6-phosphate negative feedback loop in which an increasing d-glucose-6-phospate concentration overflows, via Tps1, into d-trehalose-6-phosphate. The latter inhibits Hxk2 [38–40], the main expressed hexokinase at high d-glucose concentrations [41]. In d-glucose metabolism the negative feedback loop is essential since in the conversion of d-glucose to d-glucose-6-phosphate a single ATP molecule is consumed. Without rate control the ATP concentration would rapidly decrease to lethal levels. This phenomenon is observed in Tps1 deletion strains that lack the feedback mechanism [42, 43]. Since there is no (known) negative feedback loop in d-xylose consumption, or more specifically in the conversion of d-xylulose to d-xylulose-5-phosphate via Xks1, we hypothesize that, at high d-xylose concentrations, the ATP levels decrease significantly causing growth inhibition. To test this hypothesis, the ATP levels were analyzed in the IMX730△H strain at various d-xylose concentrations. The IMX730△H strain was grown for 16 h in mineral medium containing 0.5% d-xylose and diluted to an OD_600_ of 0.2 whereupon various concentrations of d-xylose (up to 8%) were added and ATP levels were measured in time. Compared to the 1% d-xylose control, ATP levels were significantly reduced 20 min after the addition of 4% or 8% d-xylose with 28.8 ± 5.2% and 50.0 ± 4.8%, respectively. Although after ~ 1 h, the ATP levels at high d-xylose concentration recovered to some extent they remained significantly lower as compared to the levels in cells grown on low d-xylose concentrations (Fig. 2). Reduced ATP levels, after the addition of high concentrations of d-xylose, were also observed in the IMX730-Hxk2 strain (Additional file 1: Figure S2), but did not occur when d-xylose was replaced by d-glucose (data not shown). The above observations are consistent with a substrate accelerated cellular death mechanism in which high concentrations of d-xylose cause a rapid depletion of the cellular ATP pool.

### Controlled expression of Xks1 in d-xylose metabolizing strains

**Fig. 3:**
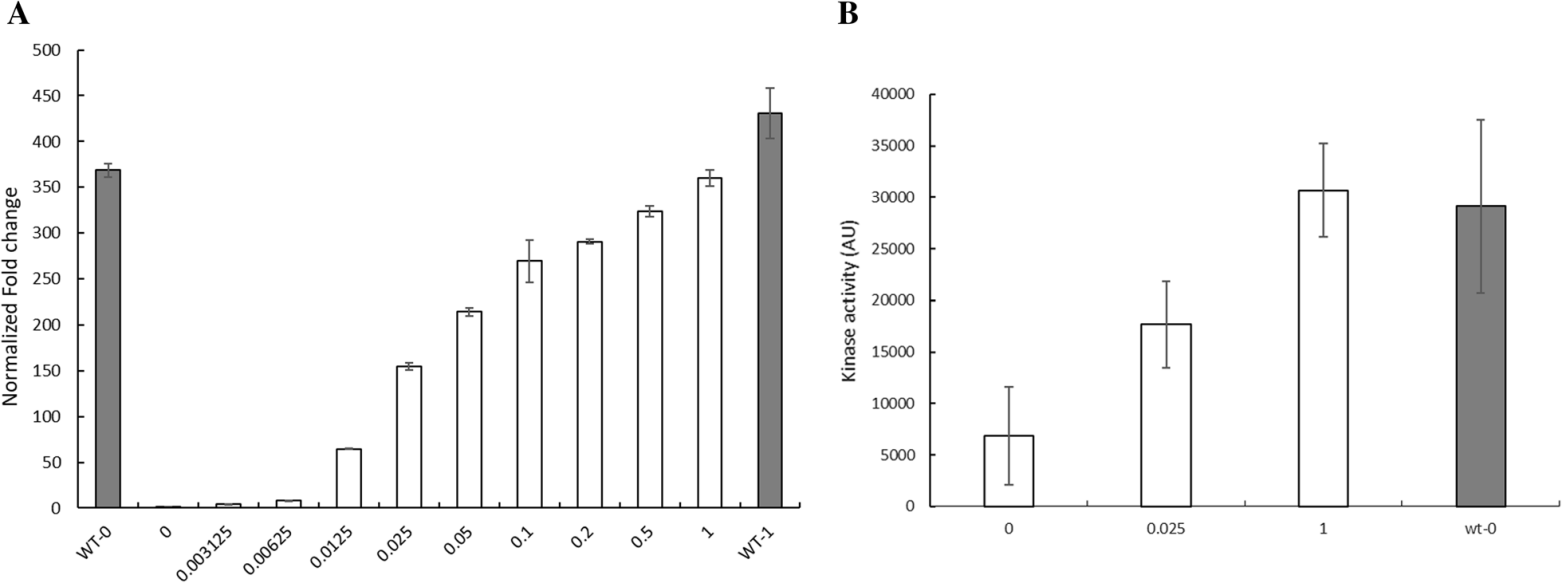
**A** Transcript fold change levels of *XKS1* in IMX730-△H (grey bars) and IMX730-pGAL::XKS1 (white bars) at various galactose concentrations ranging from 0 to 1%. WT-0 represented IMX730-△H was incubated in the absence of galactose, while WT-1 represented IMX730-△H incubated in the presence of 1% galactose. Cells were incubated aerobically in MM containing 0.5% d-xylose and 2 h after the addition of the galactose RNA was isolated. **B** Xks1 activity in IMX730-pGAL::XKS1 (white bars) at 0, 0.025 and 1% galactose and in IMX730-△H (grey bar, 0% galactose). All error bars were obtained from biological duplicates

A key ATP-utilizing step in d-xylose metabolism is the phosphorylation of d-xylulose by Xks1. The IMX730△H strain contains two copies of the *XKS1* gene: (1) the native gene, on chromosome VII, which is low expressed, and (2) the constitutively expressed gene using the strong *TEF1* promotor [44] which is integrated in the *CAN1* locus on chromosome V [15]. To control the conversion rate of d-xylulose to d-xylulose-5-phosphate via Xks1, the Tef1 promoter was replaced with the galactose inducible Gal10 promotor [45] yielding IMX730-pGAL::XKS1. IMX730-pGAL::XKS1 was inoculated in mineral medium containing 0.5% d-xylose and grown for 16 h. Cells were subsequently diluted in the same medium to an OD_600_ of 0.5 and incubated for 2 h with various concentrations of galactose ranging from 0 to 1%. Firstly, to analyze the galactose inducibility of the IMX730-pGAL::XKS1 strain in which *GAL1* is replaced by *CAS9*, the expression of Gal2 upon the addition of various galactose concentration was analyzed. Although galactose inducibility is, in principle, only affected by deletion of Gal4 (reviewed by Lohr, Venkov and Zlatanova [46]), we analyzed Gal2 expression as a marker for the galactose inducibility. The galactose transporter Gal2 showed a linear increasing expression level, up to 2400 ± 28 fold at 1% galactose as compared to no galactose (Additional file 1: Figure S3). Subsequently, the expression of *XKS1* was analyzed in IMX730-pGAL::XKS1 and in IMX730△H as the control strain. The expression of *XKS1* in the IMX730△H strain is, as expected, not altered upon the addition of 1% galactose. However, in the IMX730-pGAL::XKS1 strain the galactose concentration is directly proportional to the expression of *XKS1* in which, at 1% galactose, the expression level is comparable with that of the overexpression of *XKS1* in the IMX730△H strain (Fig. 3A). Most likely due to the low expression level of the native *XKS1* copy, a 361 ± 9 fold change at 1% galactose was obtained (Fig. 3A), which is a significant range but lower as compared to *GAL2* (Additional file 1: Figure S3). These data demonstrate that the expression of *XKS1* in strain IMX730-pGAL::XKS1 is nearly proportional to the concentration of galactose allowing us to directly examine the impact of Xks1 on d-xylose metabolism.

To show that the galactose induced expression of *XKS1* results in an expected increase in Xks1 kinase activity, d-xylulose dependent ATP consumption was measured in cell free extracts (CFEs) of IMX730△H and IMX730-pGAL::XKS1. Herein, cells were incubated for 3 h in MM with 0.5% ethanol and either 0, 0.025 and 1% galactose. Next, a cell free extract (CFE) was prepared that was incubated for 20 min with and without 1 mg/mL d-xylulose and 0.1 mM ATP. Since the Kinase Assay Kit of Sigma Aldrich measures the consumption of ATP (to ADP) by all expressed kinases, the background level of ATP consumption in the absence of d-xylulose was significant. However, upon the addition of d-xylulose, elevated kinase activity could be detected that, as compared to the expression data (Fig. 3A), correlated to the amount of the inducer galactose added (Fig. 3B). When CFEs without the addition of d-xylulose was used as background, the Xks1 activity in IMX730-pGAL::XKS1 in the presence of 0.025 and 1% d-galactose increased with 2.59 ± 0.24 and 4.49 ± 0.15 -fold, respectively. The fold-increase in Xks1 activity at 1% galactose (Fig. 3B) was significantly lower as compared to the increase in expression of *XKS1* (Fig. 3A) which can be attributed to the high kinase background levels and variation in measurements, which is evident from the large error bars. Like the expression data, the kinase activity at 0.025% galactose amounts to about 50% of that with 1% galactose. The Xks1 activity of IMX730-pGAL::XKS1 in the presence of 1% galactose was comparable with that of the IMX730△H strain (Fig. 3B) and was not affected by the galactose concentration (data not shown).

**Fig. 4:**
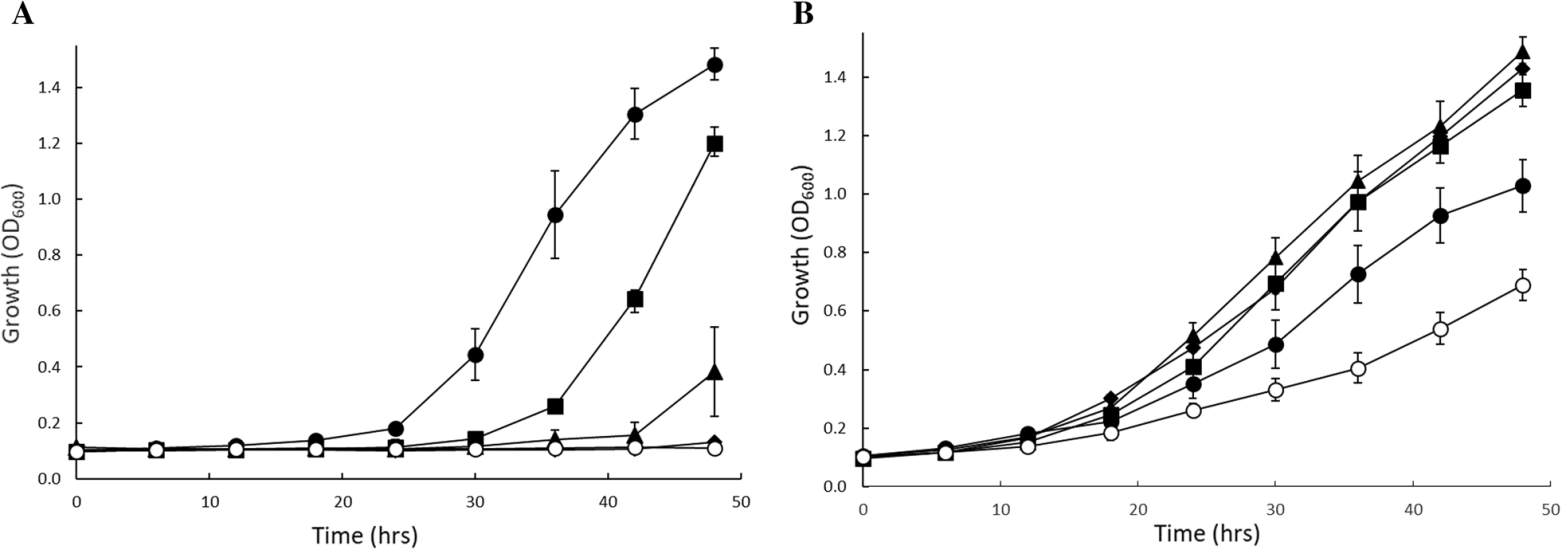
Aerobic growth in 96 wells micro-titer plates of IMX730-△H (**A**) and IMX730-pGAL::XKS1 (**B**) in mineral medium containing 0.5% d-xylose (●), 1.0% d-xylose (■), 2.0% d-xylose (▲), 4.0% d-xylose (◆) and 8.0% d-xylose (○) complemented with 0.0125% galactose. Error bars were obtained from biological triplicates

The growth of strain IMX730-pGAL::XKS1 after 24 h in mineral medium containing 8% d-xylose was analyzed with various galactose concentrations (data not shown). A concentration of 0.0125% galactose yielded optimal growth rates. Under these conditions, the expression of *XKS1* was only 64.7 ± 2.8 fold upregulated relative to no addition of galactose (Fig. 3). Therefore, 0.0125% galactose was used in an aerobic growth experiment with IMX730△H and IMX730-pGAL::XKS1, using different concentrations of D-xylose. IMX730△H showed similar growth in the 96 wells plate as compared to the shake flask experiment (Fig. 1): growth rates and biomass accumulation were significantly decreased upon the addition of increasing d-xylose concentrations in which 8% d-xylose (and in 96 wells plates also 4% d-xylose) yielded no visible growth (Fig. 4A). In contrast, the IMX730-pGAL::XKS1 strain not only showed a reduced lag phase as compared to the IMX730 strain but was also able to grow at all d-xylose concentrations tested, even at 8% d-xylose albeit with some growth inhibition. Thus, growth was significantly improved as compared to the IMX730△H strain. This data showed that reduced *XKS1* expression levels improve growth rates and biomass accumulation at high d-xylose concentrations.

**Fig. 5:**
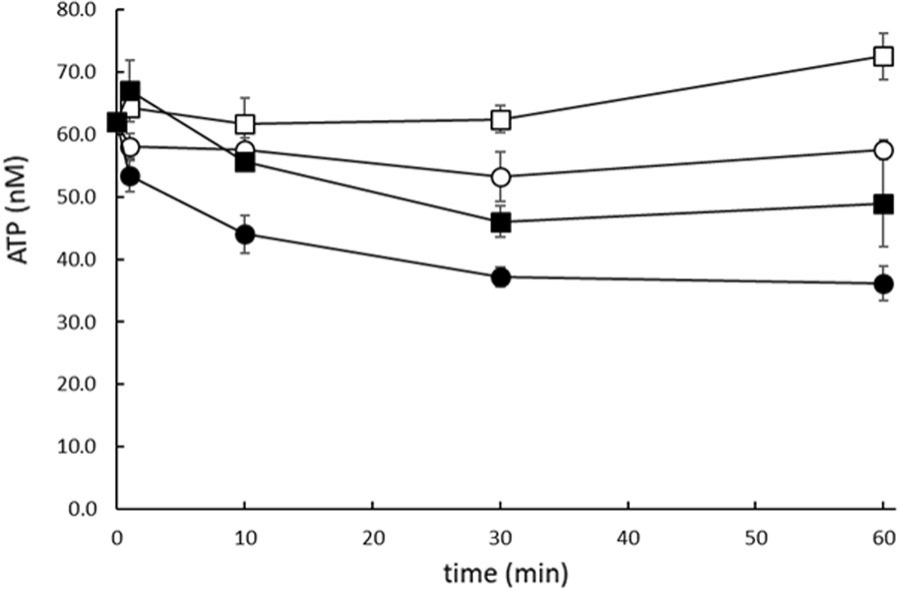
Intracellular ATP analysis in the IMX730-pGAL::XKS1 strain which was pre-incubated for 2 h with 0.003125% galactose (open symbols) or 1.0% galactose (closed symbols). 0.5% d-xylose (squares) or 8% d-xylose (circles) was added to the cultures at T0 and ATP levels were analyzed after 1, 10, 30 and 60 min. Error bars were obtained from biological duplicates

The IMX730-pGAL::XKS1 was also subjected to intracellular ATP level measurements using the conditions described above for the IMX730 strain, except that 0.003% (low *XKS1* expression) or 1% (high *XKS1* expression) galactose was included to induce the expression of *XKS1*. In IMX730-pGAL::XKS1, at a low d-xylose concentration of 0.5% (Fig. 5; squares), the measured ATP levels were higher as compared to 8% d-xylose although a significant increase was measured if *XKS1* was expressed at a low level (with 0.003% galactose, open squares). Similar to the decreased ATP levels in the IMX730 strain (Fig. 2), the ATP levels after the addition of 1% galactose (in the pre-culture) and 8% d-xylose, were decreased with 42.1 ± 6.1% in IMX730-pGAL::XKS1. When the expression of *XKS1* was significantly decreased, using 0.003% galactose, the ATP levels were 42% higher as compared to 1% galactose (Fig. 5; circles). The data show that the decreased expression of *XKS1* is accompanied by significantly increased ATP levels (already after 10 min) thereby alleviating the substrate accelerated death observed at high *XKS1* expression levels.

## Discussion

In the development of *S. cerevisiae* for second generation ethanol production there is a continued need for high-performance d-xylose metabolizing strains. Irrespective of the engineering strategy used, d-xylose metabolizing strains depend on the expression of Xks1 which converts d-xylulose into d-xylulose-5-phosphate at the expense of one ATP. Since this is the committing step in d-xylose metabolism, regulation of *XKS1* would potentially be required. However, d-xylose mediated regulation in *S. cerevisiae* does not appear to exist, likely because this sugar is not recognized as carbon source. Remarkably, also in the naturally d-xylose metabolizing yeast *Pichia stipitis*, the expression of *XKS1* (or *XYL3*) is not regulated by the d-xylose concentration [47]. In contrast, the conversion of d-glucose to d-glucose-6-phosphate by hexokinase is highly regulated in *S. cerevisiae* through gene expression [48], protein degradation [49–51] and negative feedback loops [38, 52]. A major regulatory role in d-glucose metabolism is fulfilled by Hxk2, which not only catalyzes the phosphorylation of d-glucose into d-glucose-6-phosphate, but that is also required for the glucose-induced repression of several genes, including *HXK1* and *GLK1*, and for glucose-induced expression *HXK2* itself [41, 53, 54]. The rate of d-glucose phosphorylation is also determined by a negative feedback loop thereby limiting the amount of ATP being consumed at high d-glucose availability. Accumulation of d-glucose-6-phosphate results in increased d-trehalose-6-phosphate levels, produced by the trehalose pathway, which decreases through direct inhibition the phosphorylation of d-glucose by Hxk2. Therefore the deletion of Tps1, which converts d-glucose-6-phosphate into d-trehalose-6-phosphate, is lethal for strains grown on d-glucose which has been attributed to ATP depletion [42, 43]. Hence, the overexpression of Xks1 combined with the absence of a negative feedback loop could, at high d-xylulose concentrations, potentially lead to rapid ATP consumption and cause substrate accelerated death. Such a phenomenon was observed with d-glucose conversion by Hxk2 which, when the negative feedback loop was deleted (*TPS1*), showed substrate accelerated death since all d-glucose is instantaneously converted in to d-glucose-6-phosphate thereby draining all ATP [42, 55, 56]. Likewise, reduced activity of l-ribulokinase, converting l-ribulose into l-ribulose 5-phosphate with the consumption of one ATP, is also crucial for efficient l-arabinose utilization in a l-arabinose consuming *S. cerevisiae* strain [57].

Previous studies [58, 59] showed that in metabolically engineered *S. cerevisiae* strain, only moderate transcript levels of *XKS1* are required for improved d-xylose consumption which is in agreement with Latimer et al. [60]. However, increased Xks1 expression in a strain without PPP overexpression, causes an increase in d-xylulose consumption [61] which is in contrast to Rodriguez-Peña et al. [62] who showed that the overexpression of *XKS1* in a wild-type *S. cerevisiae* strain is lethal when cells are grown solely on d-xylulose. The difference between the latter two studies can be attributed to: (1) the promoter driving *XKS1*, (2) copy number of the constructs, (3) strain usage and (4) D-xylulose concentration used. Richard et al. used d-xylulose in combination with a higher non-fermentable concentration of d-xylose, which interferes, in *Fusarium oxysporum,* with d-xylulose uptake [63], thereby affectively reducing the intracellular d-xylulose concentration. Furthermore, Ni et al. [64] and van Vleet et al. [65] showed that, via the deletion of *PHO13*, increased expression of TAL1 improved d-xylose consumption at high d-xylose concentrations. This is most likely due to increased flux through the PPP thereby increasing the ATP production downstream in the pathway.

Overall, all these results point at a phenomenon of substrate accelerated death with d-xylose as substrate, but the hypothesis of ATP depletion was not further experimentally tested nor where conditions explored where the phenomenon does not occur. Here, we show that decreased expression of *XKS1,* using the galactose tunable expression system (Fig. 3), improves growth at high d-xylose concentrations (Fig. 4B) in a xylose consuming strain. In the wild-type IMX730 strain, already at 2% d-xylose, growth rate reduction could be observed (Figs. 1, 4A) which is accompanied with decreased ATP levels (Fig. 2). At higher d-xylose concentrations, these effects are further exacerbated, resulting in near to complete growth arrest at 8% d-xylose (Fig. 2). ATP levels in *S. cerevisiae* were previously studied under various conditions (or inhibitors) and it was shown that even during starvation (either carbon or nitrogen) the ATP levels drops only up to ~ 50% [66–70]. Moreover, Takaine et al. recently showed that the stable maintenance of ATP is essential for proteostasis and that ATP levels remain remarkably stable throughout different growth phases [71]. We show that ATP levels decrease to similar levels as observed previously in starving cells, and therefore conclude that the fast conversion of d-xylulose to d-xylulose-5-phosphate by Xks1 drains the intracellular ATP levels in the cells. The current data underscores previous observations that a moderate expression of Xks1 improves the d-xylose consumption [58, 60], however in those studies a direct link to ATP depletion was not demonstrated. Wahlbom et al. showed that *XKS1* was upregulated in an evolutionary engineering experiment with a strain expressing the XK/XRD pathway and grown on 2% d-xylose [72]. The abovementioned data shows that conditions, strain and pathway determine the outcome of the optimal expression level of Xks1. Thus with a defined feedback mechanism of the regulation of the metabolic flux through Xks1, external interference is necessary to realize the optimal balance between the metabolic flux and the availability of ATP. Further, engineering of the d-xylose pathway appears not nearly as efficient as glycolysis and still requires fine-tuning either in terms of gene expression, protein degradation or the engineering of a d-xylose sensing system in *S. cerevisiae* [73, 74]. Ideally, this would mean that a demand-dependent expression of *XKS1* may be employed to maintain a high flux of d-xylose metabolism during the fermentation until all d-xylose is utilized. This would require a flexible *XKS1* expression system based on a genetic circuit that is composed of a promoter that senses d-xylose at low concentrations (comparable to the promoters of Hxt6/7) and a d-xylose sensing system based on *e.g.* Rgt2/Snf3. Both aspects, d-xylose promotors [75, 76] and d-xylose sensing [77], still requires more research and implementation to yield an economical feasible second generation biofuel process.

## Supplementary Information

**Additional file 1: Figure S1.** Aerobic growth of the IMX730△H strain complemented with the pRS313-P7T7-Hxk2 plasmid in mineral medium containing d-xylose (A) or d-glucose (B) at 0.25% (●), 0,5% (■), 2.0% (◆), and 8.0% (○) sugar. **Figure S2. **Intracellular ATP analysis after adding 0.5% d-xylose (●), 1.0% d-xylose (■), 2.0% d-xylose (▲), 4.0% d-xylose (◆) and 8.0% d-xylose (○) in the IMX730△H strain complemented with the pRS313-P7T7-Hxk2 plasmid. The IMX730△H-Hxk2 pre-culture was grown aerobically for 16 hours in minimal medium supplemented with 0.5% d-xylose. Error bars were obtained from biological duplicates.** Figure S3.** Transcript fold change levels of *GAL2* in IMX730-pGAL::XKS1 at various galactose concentrations ranging from 0% to 1%. Cells were incubated aerobically with 0.5% d-xylose and 2 hours after the addition of the galactose RNA was isolated. Error bars were obtained from biological duplicates. **Table S1. **Strains and plasmids used in this study. **Table S2.** Oligonucleotides used in Cas9 related deletion or integration. **Table S3.** Oligonucleotides RT-PCR.

## Data Availability

The datasets supporting the conclusions of this article are included within the article or the additional file (Additional file 1: Figs. S1, S2 and S3; Tables S1, S2 and S3). Strain IMX730 can be requested from Jack T. Pronk.

## Abbreviations

ATP: Adenosine tri-phosphate
FC: Fold change
Hxk: Hexokinase
Hxt: Hexose transporter
MM: Mineral medium
OD: Optical density
PPP: Pentose phosphate pathway
Xks1: Xylulose kinase
XI: Xylose isomerase
