# Supplementary material for: d-xylose accelerated death of pentose metabolizing Saccharomyces cerevisiae
Source: Biotechnol Biofuels Bioprod. 2023 Apr 17;16:67. doi: 10.1186/s13068-023-02320-4 (PMC10111712; doi:10.1186/s13068-023-02320-4)
Supplement: Supplementary file 1 — Additional file 1: Figure S1. Aerobic growth of the IMX730△H strain complemented with the pRS313-P7T7-Hxk2 plasmid in mineral medium containing d-xylose (A) or d-glucose (B) at 0.25% (●), 0,5% (■), 2.0% (◆), and 8.0% (○) sugar. Figure S2. Intracellular ATP analysis after adding 0.5% d-xylose (●), 1.0% d-xylose (■), 2.0% d-xylose (▲), 4.0% d-xylose (◆) and 8.0% d-xylose (○) in the IMX730△H strain complemented with the pRS313-P7T7-Hxk2 plasmid. The IMX730△H-Hxk2 pre-culture was grown aerobically for 16 hours in minimal medium supplemented with 0.5% d-xylose. Error bars were obtained from biological duplicates. Figure S3. Transcript fold change levels of GAL2 in IMX730-pGAL::XKS1 at various galactose concentrations ranging from 0% to 1%. Cells were incubated aerobically with 0.5% d-xylose and 2 hours after the addition of the galactose RNA was isolated. Error bars were obtained from biological duplicates. Table S1. Strains and plasmids used in this study. Table S2. Oligonucleotides used in Cas9 related deletion or integration. Table S3. Oligonucleotides RT-PCR. [file 13068_2023_2320_MOESM1_ESM.docx]

## D-xylose accelerated death of pentose metabolizing *Saccharomyces cerevisiae*

## Jeroen G. Nijland, Xiaohuan Zhang and Arnold J.M. Driessen

**
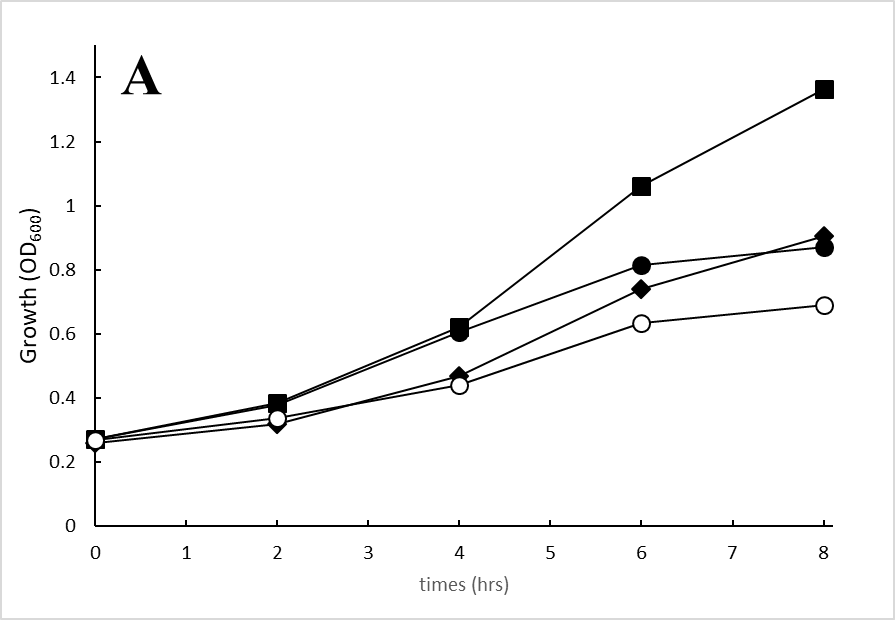
Additional file data**

**
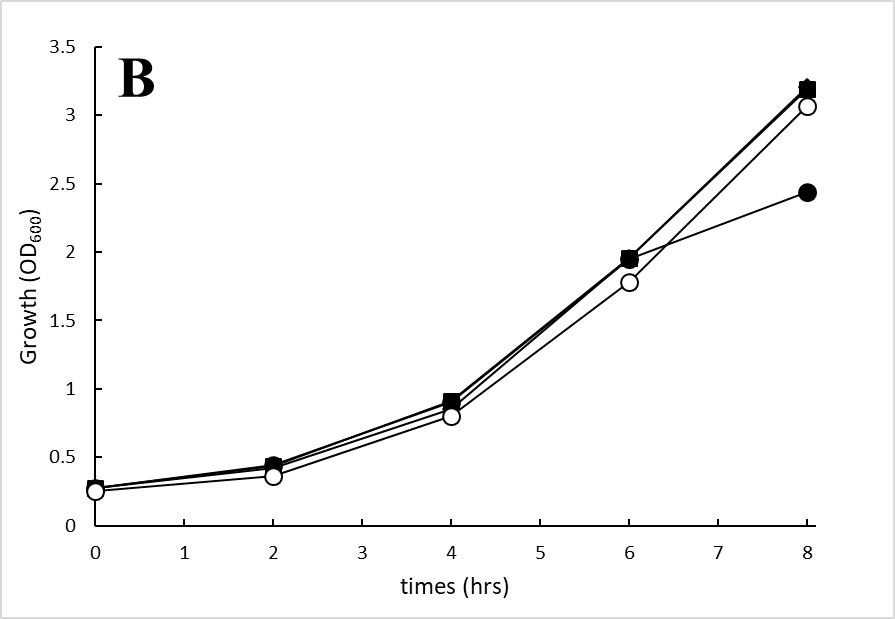
**

**Figure S1.** Aerobic growth of the IMX730△H strain complemented with the pRS313-P7T7-Hxk2 plasmid in mineral medium containing D-xylose (A) or D-glucose (B) at 0.25% (⚫), 0,5% (⯀), 2.0% (⯁), and 8.0% (⭘) sugar.


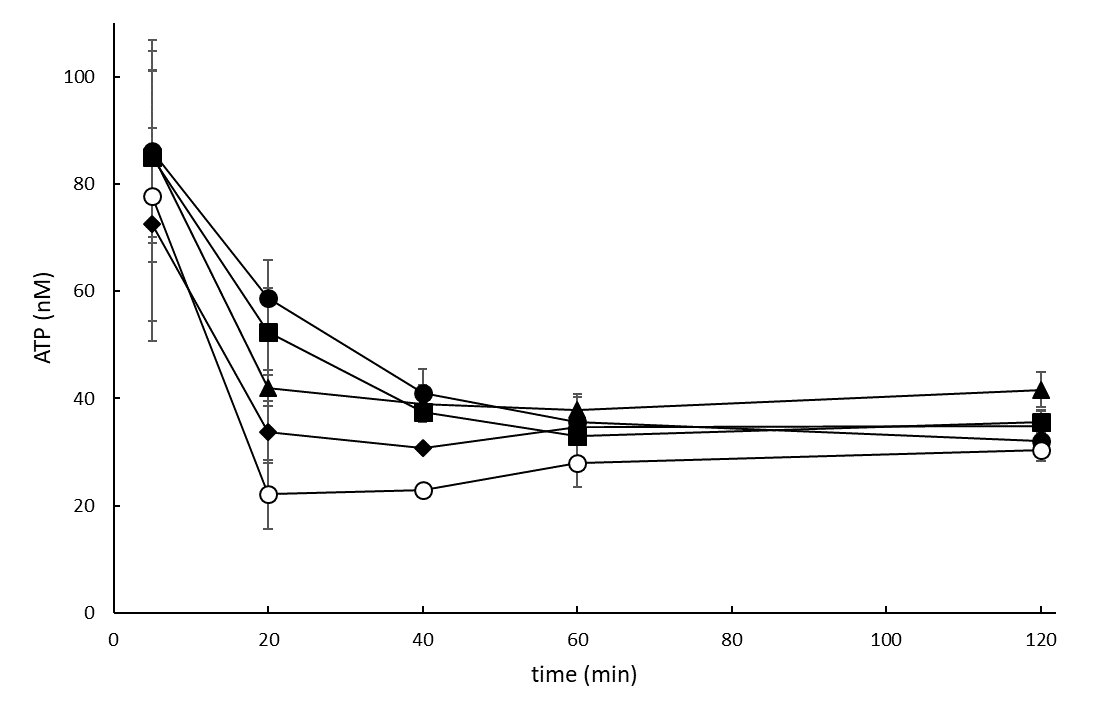


**Figure S2.** Intracellular ATP analysis after adding 0.5% D-xylose (⚫), 1.0% D-xylose (⯀), 2.0% D-xylose (▲), 4.0% D-xylose (⯁) and 8.0% D-xylose (⭘) in the IMX730△H strain complemented with the pRS313-P7T7-Hxk2 plasmid. The IMX730△H-Hxk2 pre-culture was grown aerobically for 16 hours in minimal medium supplemented with 0.5% D-xylose. Error bars were obtained from biological duplicates.


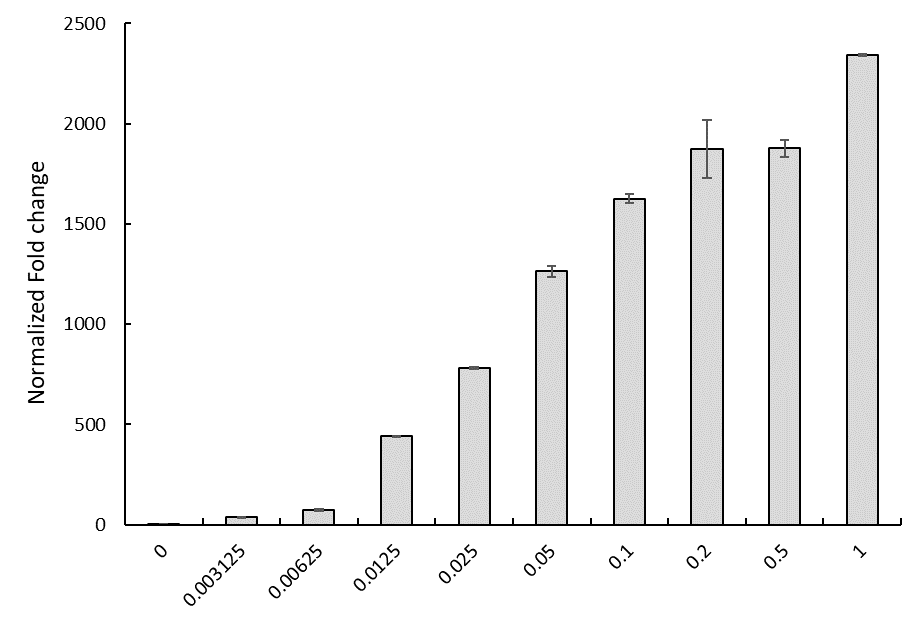


**Figure S3.** Transcript fold change levels of *GAL2* in IMX730-pGAL::XKS1 at various galactose concentrations ranging from 0% to 1%. Cells were incubated aerobically with 0.5% D-xylose and 2 hours after the addition of the galactose RNA was isolated. Error bars were obtained from biological duplicates.

**Table S1. Strains and plasmids used in this study**

| Strain/plasmid | Relevant genotype and/or characteristics | Source or reference |
| --- | --- | --- |
| Strains |  |  |
| IMX730 | MATa ura3-52 his3-1 leu2-3112 MAL2-8c SUC2 glk1::*Sp*his5, hxk1::KlLEU2 gal1::cas9- amdS gre3::pTDH3 RPE1 pPGK1 TKL1, pTEF1 TAL1 pPGI1 NQM1 pTPI1 RKI1 pPYK1 TKL2 can1::(pTPI xylA tCYC)∗9 pTEF1-XKS1 hxk2::*Pc*araT | (1) |
|  |  |  |
| IMX730△H | IMX730 ::*Sp*his5 | This study |
| IMX730-pGAL::XKS1 | IMX730△H pGAL *XKS1* | This study |
| Plasmids |  |  |
| pMel10 | *E. coli*/yeast vector, uracil autotrophic marker | (2) |
| pMel10-His5  pMel16 | pMel10, expressing sgRNA His5  *E. coli*/yeast vector, histidine autotrophic marker | This study  (2) |
| pMel16-Pgal10 | pMel16, expressing sgRNA linker pTEF1 and XKS1 | This study |

|  |  |
| --- | --- |

**Table S2.** Oligonucleotides used in Cas9 related deletion or integration.

| Name | Sequence (5’ 🡪 3’) |
| --- | --- |
| F Tar *sp*His5  R Tar *sp*His5  F Rep *sp*His5  R Rep *sp*His5  F chk KO *sp*His5  R chk KO *sp*His5  F tar Pgal10  F tar Pgal10  F Rep Pgal10  R Rep Pgal10  F chk Gal10  R chk Xks1 | TGCGCATGTTTCGGCGTTCGAAACTTCTCCGCAGTGAAAGATAAATGATCAATTCCAGCTGCTACCGAAAGTTTTAGAGCTAGAAATAGCAAGTTAAAATAAGGCTAGTCCGTTATCAAC  GTTGATAACGGACTAGCCTTATTTTAACTTGCTATTTCTAGCTCTAAAACTTTCGGTAGC  AGCTGGAATTGATCATTTATCTTTCACTGCGGAGAAGTTTCGAACGCCGAAACATGCGCACGCA  CGCCCATATAAATATCCGCTATCAACAGAACCCCCAACCCCCCCATCAGTGCCCAACTCAACTTACCATCTTCTCTCCTTTATATATATATATATATATGTATATTTTCAGTGTATATAC  GTATATACACTGAAAATATACATATATATATATATATATAAAGGAGAGAAGATGGTAAGTTGAGTTGGGCACTGATGGGGGGGTTGGGGGTTCTGTTGATAGCGGATATTTATATGGGC  ACGCGGCGCTTGCACCCCGC  GTGTCATTGTTCAACACGTATGCG  TGCGCATGTTTCGGCGTTCGAAACTTCTCCGCAGTGAAAGATAAATGATCCAGCATACTTCGGGAACCGTGTTTTAGAGCTAGAAATAGCAAGTTAAAATAAGGCTAGTCCGTTATCAAC  GTTGATAACGGACTAGCCTTATTTTAACTTGCTATTTCTAGCTCTAAAACACGGTTCCCGAAGTATGCTGGATCATTTATCTTTCACTGCGGAGAAGTTTCGAACGCCGAAACATGCGCA  TGAAAACCTTGCTTGAGAAGGTTTTGGGACGCTCGAAGGCTTTAATTTGCACGGATTAGAAGCCGCCGAGC  ATTGTGTTGGAAACCTCTCTTGTCTGTCTCTGAATTACTGAACACAACATGGTTTTTTCTCCTTGACGTTAAAGTA  TGGGGTAATTAATCAGCGAAGCG  CTAGAGCCTCTAACCACATGGC |

- Underlined represents the gene specific target

**Table S3.** Oligonucleotides RT-PCR.

| Name | Sequence (5’ 🡪 3’) |
| --- | --- |
| F Act1  R Act1  F Gal2  R Gal2  F Xks1  R Xks1 | GGATTCTGAGGTTGCTGCTTTGG  GAGCTTCATCACCAACGTAGGAG   \| \| TCAATGGAGAGTTCCATTAGGGC \| \| --- \| \| CCAGAGGATCCTGCCGTCCAG \| \| \| --- \| --- \| --- \| \| GGGTCCTGCCAGCAGCACGG  CTTTGCAGTACTGTGGTCTTGCC \| |

1. Verhoeven MD, de Valk SC, Daran J-MG, van Maris AJA, Pronk JT. Fermentation of glucose-xylose-arabinose mixtures by a synthetic consortium of single-sugar-fermenting Saccharomyces cerevisiae strains. FEMS Yeast Res [Internet]. 2018 Dec 1 [cited 2019 Jan 28];18(8). Available from: https://academic.oup.com/femsyr/article/doi/10.1093/femsyr/foy075/5054444

2. Mans R, van Rossum HM, Wijsman M, Backx A, Kuijpers NGA, van den Broek M, et al. CRISPR/Cas9: a molecular Swiss army knife for simultaneous introduction of multiple genetic modifications in Saccharomyces cerevisiae. FEMS Yeast Res [Internet]. 2015 Mar 19 [cited 2016 Nov 21];15(2):fov004–fov004. Available from: http://femsyr.oxfordjournals.org/cgi/doi/10.1093/femsyr/fov004
